# Effects of High Intensity Interval Training on Executive Function in Children Aged 8–12 Years

**DOI:** 10.3390/ijerph16214127

**Published:** 2019-10-26

**Authors:** Nobuaki Tottori, Noriteru Morita, Kenji Ueta, Satoshi Fujita

**Affiliations:** 1 Faculty of Sport and Health Science, Ritsumeikan University, Kusatsu 525-8577, Japan; sh0037xp@ed.ritsumei.ac.jp (N.T.); ueta@fc.ritsumei.ac.jp (K.U.); 2 Department of Cultural Studies, Hokkaido University of Education, Iwamizawa 068-8642, Japan; morita.noriteru@i.hokkyodai.ac.jp

**Keywords:** primary school-aged children, high intensity interval training, cardiorespiratory endurance, muscular endurance, working memory, planning

## Abstract

This study investigated the effects of a high intensity interval training (HIIT) program on both physical fitness and executive functions in children. Fifty-six children aged 8–12 years participated in this study, and were divided into a HIIT group and a control group. The HIIT group performed three sessions of the 8- to 10-min HIIT program per week for 4 weeks. Before and after the intervention, 20-m shuttle runs, sit-ups, and standing long jumps were assessed as test of physical fitness. In addition, the executive function was assessed using the digit span forward (DSF) test, digit span backward (DSB) test, and Tower of Hanoi test. Only the HIIT group experienced significant improvement when completing the 20-m shuttle run (*p* = 0.042) and sit-ups (*p* < 0.001). Regarding executive function, the number of correct answers in DSB test significantly increased only in the HIIT group (*p* = 0.003). However, the standing long jump, DSF, and the Tower of Hanoi test performance did not change after intervention. The findings of the present study suggest that HIIT has positive effects on a core executive function such as working memory in addition to components of the physical fitness such as cardiorespiratory endurance and muscular endurance.

## 1. Introduction

Recent studies have suggested that higher levels of physical fitness, especially aerobic fitness and sufficient physical activity, are linked to change in the brain structure and function, such as cognitive function and academic achievement in children [1,2,3,4]. Executive function develops as a child’s brain matures, and plays an important role in the cognitive, behavioral, and social-emotional development of children [5]. Considering that the achievement rate of the recommendation level of physical activity and physical fitness in children has been decreasing [6,7], an increase in aerobic fitness is necessary to improve a wide range of health parameters, including executive functions.

Executive functions refer to a subset of top-down cognitive control processes for goal-directed behavior [8]. The core executive functions consist of working memory (the ability to hold information in mind and manipulate it), inhibition (the ability to control one’s attention, behavior, thoughts, and/or emotions), and cognitive flexibility (the ability to switch between thinking and to change perspectives spatially) [8]. In addition, the higher-order executive functions of planning and problem-solving are built from those core executive functions [8]. Planning is a component that has been directly linked to the process of organizing a sequence of operations to achieve a final goal. Executive functions provide the foundation for academic abilities such as reading, comprehension, and mathematical problem solving [9,10]. Particularly, in primary school-aged children, working memory performance has been shown to predict academic performance such as the performance in mathematics [11]. In addition, working memory is related to dual task performance in life activity [12]. Therefore, working memory has important roles not only in academic performance, but also in everyday life.

Most previous cross-sectional studies reported that aerobic fitness is related to inhibition [1,13,14] and working memory [13,15,16] in children. A previous study suggested that children with higher levels of aerobic capacity have greater working memory scores than do children with lower levels of aerobic capacity [4]. In addition, aerobic capacity is associated with superior mathematic performance in algebraic functions [16]. Regarding the longitudinal intervention studies, a meta-analysis revealed that longitudinal aerobic and cognitively engaging activity programs have a small to moderate effect on working memory (Hedges’ g = 0.36), but no significant effect on inhibition in preadolescent children [17]. Some aerobic exercise intervention studies clarified the positive effect on working memory during preadolescence [18,19,20]. Furthermore, a theoretical review showed that working memory is the only executive function that improves with chronic exercise but not in response to acute exercise in children [21]. These findings suggested that aerobic fitness and exercise have a positive effect on working memory in preadolescent children. On the other hand, few studies have considered the effect training has on higher-order executive function [20,22,23]. The results of those studies were inconsistent about the effect of training in children. Davis et al. [22] suggested that aerobic exercise has a significantly positive effect on planning skills in overweight children aged 7–11 years old. In contrast, other studies showed no effect of a physical education program consisting of aerobic activities with cognitive effort in preadolescent children [20,23]. The effects of only aerobic exercise on planning in normal-weight children remain unexplored. Therefore, it is necessary to clarify the effect of aerobic fitness on planning skills in children.

High intensity interval training (HIIT) is now acknowledged as a potent training modality for increasing aerobic fitness [24,25] and mental health [26] in children, although HIIT can be completed within a shorter period compared to traditional continuous aerobic training. Since the unit of physical education and/or the time of physical activity diminishes in school, children in school require short-term exercises that effectively improve physical fitness [27]. Therefore, HIIT may be adequate for improving aerobic fitness in the younger population. Costigan et al. [26] indicated that the HIIT program could potentially enhance the cognitive flexibility in adolescents. In addition, Eather et al. [28] indicated that the HIIT program significantly improved cognitive flexibility and that a change in aerobic capacity is associated with changes in the scores of cognitive flexibility test in young adults. Therefore, the HIIT program might not potentially improve only aerobic fitness but also executive function in primary school-aged children. Only one study [29] reported the effects of a 6-week high intensity training on working memory and inhibitions. However, they did not assess physical fitness or executive function, including higher-order executive function (i.e., planning), in children younger than 14 years. In first year junior high school students (aged 12–13 years), body mass index and cardiorespiratory endurance are related to academic achievement [30,31]. Therefore, higher aerobic fitness during elementary school may be important for executive functions as well as academic success in middle and/or high schools.

The purpose of this study was to evaluate the efficacy of HIIT for improving physical fitness including the aerobic fitness, muscular endurance and strength, and executive functions such as working memory and planning in children aged 8–12 years. We tested the hypothesis that HIIT could improve cardiorespiratory endurance, working memory, and planning.

## 2. Materials and Methods

### 2.1. Participants

Fifty-eight children in the 3rd–6th grades with no prior HIIT experience were recruited from two school districts in this study. They were allocated into two groups based on the living area, a HIIT group (19 boys and 10 girls) or a control group without training intervention (14 boys and 15 girls). Two boys in the HIIT group dropped out according to the sick during the intervention. Their maturity status was evaluated in years from the peak height velocity estimated by a maturity offset, which was derived from anthropometric data [32]. Prior to testing, the children’s legal guardians completed a health history and the Attention Deficit Hyperactivity Disorder (ADHD) Rating Scale IV [33]. All of the children did not have any medical or orthopedic issues that limited their ability to engage in exercise and measurements. They also had neither neurological diseases nor attentional disorders, and scored below the 90th percentile on the ADHD Rating Scale IV. In addition, moderate-to-vigorous intensity physical activity (MVPA) was assessed using an item from the World Health Organization Health Behavior in School-aged Children (HBSC) survey whose validity had been confirmed in Japan [34]. During the MVPA survey, children were asked how many days they were physically active for a total of 60 min per day over 7 days. Their parents were informed of the experimental procedures and provided written consent for participation in this study. Additionally, the children assented to participate. All procedures were approved by the Ethics Committee of Ritsumeikan University (BKC-IRB-2018-013). Prior to starting the present study, power analysis was performed (G*Power 3; Heinrich-Heine-Universität, Düsseldorf, Germany) to calculate the adequate sample size (F-test, effect size = 0.25, α error = 0.05, power = 0.80). According to this calculation, 24 participants were required.

### 2.2. HIIT Intervention

Children in the training group participated in the HIIT program at a gymnasium of the university during the summer vacation of 2018, and performed three sessions of the HIIT per week over four weeks for a total of 12 sessions. There was a minimum recovery of 48 h between sessions. Sessions lasted from 8–10 min (1st–4th session, 8 min; 5th–8th session, 9 min; 9th–12th session, 10 min) with exercise to rest ratios of 30 s:30 s. The HIIT program consisted of aerobic and core exercise using one’s own weight (13 m or 26 m shuttle runs, jumping jacks, vertical jumps, mountain climbers, and plank in and out jumps). Following 10 min of warm up including jogging and dynamic stretching, children performed the exercise with maximal effort. An instructor monitored the real-time heart rate (HR), with participants wearing Polar H10 HR sensors (Polar Electro, Kempele, Finland) connected to the Polar Team iPad application (Polar Electro, Finland) throughout each main exercise. The target peak HR was set at 85% of the maximum HR (%HRmax) predicted by age “208 − (0.7 × Age)” [35] or higher to ensure the appropriate exercise intensity. At the end of each session, the participants were allowed to cool down for 5 min to reduce the HR.

### 2.3. Measurements

#### 2.3.1. Physical Fitness

Physical fitness was assessed by the 20-m shuttle run (cardiorespiratory endurance), sit-ups (muscular endurance), and standing long jump (lower limb muscular strength) according to the New Physical Fitness Test of the Ministry of Education, Culture, Sports, Science and Technology in Japan (for details, see Morita et al. [31]). All tests were performed at a gymnasium or indoor hall with the children wearing shoes. Prior to the measurements, the children were instructed on the rules of the test and performed warm-ups. The number of completed laps of the 20-m shuttle run, the highest value obtained on two trials of sit-ups, and the longest distance for standing long jump were used in further analyses. This section may be divided by subheadings. It should provide a concise and precise description of the experimental results, their interpretation, as well as the experimental conclusions that can be drawn.

#### 2.3.2. Executive Function

Short-term memory and working memory were assessed using the Digit Span Forward/Backward test (forward, DSF; backward, DSB) according to the fourth edition of the Wechsler Intelligence Scale for Children manual [36]. Three trials in each level of sequences were performed as the exception to the fourth edition of the Wechsler Intelligence Scale for Children. The examiner pronounced the list of digits at a rate of approximately one digit per second. Children were required to repeat the list in the same order for the DSF and in the reverse order for the DSB immediately after the examiner finished listing the digits. The tests started with a sequence length of 2 and increased to a sequence length of 9 for the DSF and to sequence length of 8 for the DSB. Three trials were performed for each sequence length. This test was discontinued when children failed to repeat three attempts at the same level. Each correct answer was scored as 1 point, so the maximum scores were 24 points for the DSF and 21 points for the DSB. The maximum span was established as the longest level of sequences of two correct answers out of three trials at the same level.

Planning skills was assessed using the traditional wooden version of the Tower of Hanoi (ToH) [37]. This test required that differently sized disks be moved across three pegs according to a picture depicting the goal layout with all disks at the right peg as quickly and with as few moves as possible. Children had to adhere to three rules during trials: (1) only one disk could be moved at a time, (2) a disk could not be placed on the table or held in the hand while another disk was being moved, and (3) a larger disk may not be placed on top of a smaller disk. There were three-disk, four-disk, and five-disk problems. The fewest moves were 7, 15, and 20 for the three-disk, four-disk, and five-disk problems, respectively. The test was started after providing participants with instructions and practicing with a two-disk problem. The time and number of moves were recorded during each test.

### 2.4. Statistical Analysis

Data are expressed as the mean ± standard deviation with 95% confidence intervals. An independent samples t-test was used to compare each variable between groups at the baseline. When a significant difference was observed at the baseline, analysis of covariance was used to compare the post-value between groups with adjustment for the pre-value and confounders. The effects of training were analyzed for statistical significance via two-way repeated measures analysis of variance (ANOVA) (time [pre, post] × group [HIIT, Control]). When significant group-by-time interactions were observed, group specific post hoc tests (a paired *t*-test) were used to identify statistically significant comparisons. In addition, we calculated the 95% confidence intervals of changes in variables. Effect sizes were calculated using the partial eta squared for ANOVA and Cohen’s d for post hoc tests. All statistical analyses were performed using SPSS version 25 (SPSS Inc., Chicago, IL, USA). The alpha level was set at *p* < 0.05 to indicate statistical significance.

## 3. Results

The baseline anthropometric and demographic data are presented in Table 1. There were no significant differences between both groups in terms of age, year from peak height velocity, height, weight, body mass index, and days of MVPA at the baseline. In addition, there was no change in days of MVAP from baseline to post intervention. For the comparison of baseline differences in physical fitness between groups, significant differences were observed for the 20-m shuttle run (*p* = 0.009) and standing long jump (*p* = 0.048). There was no significant difference between groups regarding executive function at the baseline. The average HR of children during the training sessions was 170.0 ± 9.4 bpm (%HRmax, 85.1 ± 4.7). The mean peak HR was 193.8 ± 8.0 bpm (%HRmax, 97.0 ± 4.0). The target HR was achieved for 98.5% of the entire exercise session.

**Figure 1:**
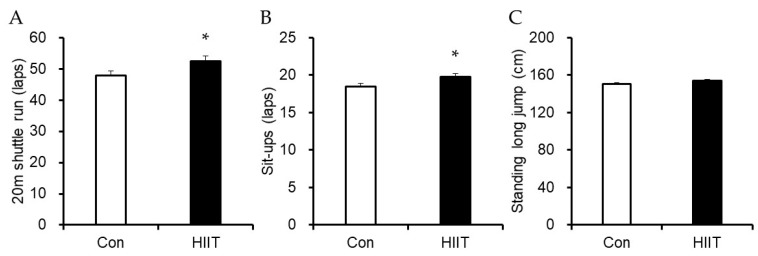
Comparisons of physical fitness via 20-m shuttle run (**A**), sit-ups (**B**), and standing long jumps (**C**), adjusted for pre-test values and age between groups. Values are presented as means ± standard deviation, adjusted for pre-test values and age. * *p* < 0.05, significant difference between groups.

Regarding physical fitness (Table 2), significant group-by-time interactions were observed for the 20-m shuttle run (F = 4.313, *p* = 0.043, η_p_^2^ = 0.074) and sit-ups (F = 4.818, *p* = 0.032, η_p_^2^ = 0.082). Post hoc analysis revealed significant increases in 20-m shuttle run (*p* = 0.042, d = 0.417) and sit-ups (*p* < 0.001, d = 0.683) in the HIIT group only. Otherwise, there was no main effect and interaction in the standing long jump. After adjusting for age and the pre-value, significantly higher values were obtained in the 20-m shuttle run and sit-ups in the HIIT group than in the control group (Mean ± SE; 20-m shuttle run: 52.51 ± 1.55 vs. 47.91 ± 1.49, *p* = 0.044; sit-ups: 19.77 ± 0.47 vs. 18.42 ± 0.46, *p* = 0.048) (Figure 1). Similar to the result obtained in the two-way ANOVA, the analysis of covariance showed no difference between the groups regarding standing long jump.

Regarding executive functions (Table 3 and Table 4), statistical analysis showed a significant group-by-time interaction for the score obtained in the DSB test (F = 4.304, *p* = 0.043, η_p_^2^ = 0.074) and a trend of interaction for the maximum span in the DSB test (F = 3.745, *p* = 0.058, η_p_^2^ = 0.065). The HIIT group showed significant improvements in the score (*p* = 0.003, d = 0.549) and the maximum span (*p* = 0.010, d = 0.539) in the DSB test. On the other hand, in the control group, there was no improvement in DSB performance. A significant time effect was observed for the score obtained in the DSF test (F = 18.430, *p* < 0.001, η_p_^2^ = 0.254), while no significant interaction was observed in the DSF test. Significant time effects were observed for the 3-disk (time: F = 28.331, *p* < 0.001, η_p_^2^ = 0.353; number of moves: F = 9.918, *p* = 0.003, η_p_^2^ = 0.160) and 4-disk (time: F = 34.158, *p* < 0.001, η_p_^2^ = 0.396; number of moves: F = 8.344, *p* = 0.006, η_p_^2^ = 0.138) versions of the ToH, while no significant interactions were observed.

## 4. Discussion

The present study aimed to investigate the effect of the HIIT program on physical fitness, working memory, and planning in children aged 8–12 years. Children in the HIIT group showed significant improvements in cardiorespiratory endurance on the 20-m shuttle run and muscular endurance on sit-ups. Regarding executive functions, we found a significant effect of HIIT on working memory measured by the DSB test and no significant improvement for planning as evaluate using ToH test.

Executive function is positively related to aerobic fitness in children [1,4,13,15,16]. An intervention study conducted by Kamijo et al. [18] indicated the positive effects of a 9 month aerobic training intervention on the Sternberg task performance (the working memory test) in children aged 7–9 years. In present study, there was a significant improvement in working memory as measured by the DSB test. Similarly, the 8-week long school-based HIIT program yielded small and moderate effects on cognitive flexibility (trail making test B) in adolescents [26]. Moreover, a previous study targeting university students examined the effects of HIIT on cognitive flexibility, and the association of changes in aerobic capacity with changes in the score obtained on the cognitive flexibility test [28]. Therefore, although causality has not been established, the result of this study corroborates those of previous studies regarding the positive effects of aerobic training (i.e., HIIT) in addition to traditional aerobic exercise (e.g., endurance running and cycling) on executive function in primary school-aged children.

While improvements in working memory were observed, this study found no significant improvement in planning skills. This result corroborated that of a previous study conducted by van der Niet et al. [20] that targeted participants in a similar age group as that of the present study (8–12 years), and reported that a 22-week physical activity program comprising moderate-vigorous-intensity activity had no effect on planning skills. In contrast to the present and a previous study [20], a positive relation was found between planning skills assessed by the Tower of London test and both total volume physical activity and moderate to vigorous physical activity in children aged 8–12 years [38]. Planning skills are part of the higher order executive function and are derived from core executive functions [8]. From these studies, planning skills could not be detected in this short-term intervention study since planning is more complex than working memory. Future studies involving larger sample sizes and long-term intervention are needed to clearly investigate the effects of physical training on planning skills.

In the present study, a 4-week HIIT program significantly improved the cardiorespiratory endurance and muscular endurance capacity compared to the control group in children. HIIT has the same capacity for improving the oxygen uptake during peak exercise (VO_2_peak) [24], although HIIT can be completed within a shorter period compared to traditional continuous training. In addition, HIIT improves the aerobic and anaerobic capacities, while endurance running training only improves aerobic capacity [39]. Therefore, HIIT may be a time-efficient and effective method for improving the cardiorespiratory capacity. We collected data regarding the HR during all exercise sessions, and confirmed them to ensure appropriate exercise intensity (≥85%HRmax). An increase in 3.15 laps (+7.4%) on the 20-m shuttle run was achieved in comparison to the pre-test score, which could be converted into an estimated increase in VO_2_peak of 0.57 mL/kg/min (+1.17%) using the equation reported in a previous study [40]. Although we could confirm the validity of the training design and significant improvement, a lower level of improvement in the aerobic capacity was obtained in the present study compared to those reported in previous studies [24,41]. Baquet et al. [24] reported that the VO_2_peak is significantly improved (+4.8%) after 7 weeks of interval training three times a week with each lasting 18–39 min, in children aged 8–11 years. Additionally, McManus et al. [41] reported that the VO_2_peak is significantly improved (5.5 mL/kg/min; +12.1%) after 8 weeks of interval training three times a week, with each session lasting 20 min in children aged 9–11 years. The exercise duration and intervention period were also lower in the present study compared to those reported in previous studies. A systematic review showed that HIIT, performed two or three times a week and with a minimum intervention duration of 7 weeks, elicits the greatest improvement in physical and cardiovascular health among children and adolescents [42]. From these previous results, the HIIT may have a larger effect of aerobic fitness in children if the program has a larger number of training sessions.

In the present study, no improvement in lower body muscular strength was observed even though the HIIT program included body weight exercises targeting the lower limbs (e.g., jumping jacks and vertical jumps). However, Baquet et al. [39] reported a significant improvement in the standing long jump (9.6%, *p* < 0.001) in children (aged 9.7 ± 0.8 years) after a 7 week HIIT program performed twice a week. Consistent with these results, a meta-analysis of studies conducted in adolescents revealed that the overall effect of HIIT on muscular fitness was not significant [43]. In addition, Eather et al. [28] reported no significant effect of an 8-week HIIT program on lower body muscular fitness in university students. These results may be due to the fact that no training load and/or a lower number of repetitions was used, which was not enough to stimulate the lower limb muscles during the training session and over the intervention period. Considering that “over load” is an important principle for improving muscular strength, body weight may be an inadequate load for performing standing long jumps. Due to the paucity of the evidence in this regard, future studies are needed to clearly examine the effect of HIIT on muscular fitness in primary school-aged children.

Present study suggests the positive effect of HIIT on the working memory of children. This result has the potent of academic success across the life span since previous studies reported that working memory is related to academic performance such as in mathematics [11]. In addition, the program of this study can be used in physical education and/or before-school physical activity to increase aerobic fitness, muscular endurance and working memory. The present study has some limitations. First, participants could not be allocated randomly into two groups. As such, this study recruited a small sample to detect a small effect size and an unequal proportion of girls and boys. The present study used the two-way ANOVA because prior analysis using three-way ANOVA (gender, time, and group effect) did not show interactions and a gender effect. In addition, no statistical different proportion between groups was confirmed using the chi-squared test. Second, only the executive functions of working memory and planning were clarified regarding their effect on HIIT in the present study. As we mentioned before, planning skills are derived not only from working memory but also from other functions such as inhibitions and cognitive flexibility [8]. Consequently, future studies should focus on the other functions. Third, we did not collect the socioeconomic status including parent education and income which are related to baseline value of executive functions. Finally, the present study used field-based methods to assess physical fitness. Regarding the assessment of aerobic fitness, evaluation of the maximum rate of oxygen consumption measured during incremental exercise (VO_2_max) by indirect calorimetry is considered to be the gold standard. For other forms of physical fitness, laboratory-based methods may detect more valid values compared to field-based methods.

## 5. Conclusions

The present study showed that a 4-week HIIT program significantly improved cardiorespiratory endurance and muscular endurance in children aged 8–12 years. In addition, there was a significant increase in working memory caused by HIIT intervention. To the best of our knowledge, this is the first study to demonstrate the positive effects of HIIT on both physical fitness and executive functions in primary school-aged children. These findings support the practical implication of HIIT being beneficial for both physical fitness and executive function in school because HIIT can be completed within a short duration (8 to 10 min in the present study). Future studies are needed to evaluate the feasibility of HIIT in school-based programs (e.g., physical education).

## Figures and Tables

**Table 1:** Participant’s baseline demographics.

Variables	Control Group			HIIT Group		
n	29 (14 boys)			27 (17 boys)		
Age (years)	10.4	±	1.1	10.0	±	1.0
Y-PHV (years)	−2.7	±	1.3	−2.1	±	1.7
Body height (cm)	139.4	±	8.5	138.7	±	10.0
Body mass (kg)	32.0	±	6.3	33.2	±	10.5
BMI (kg/m^2^)	16.3	±	1.8	16.9	±	2.7
MVPA (day/week)	2.5	±	2.0	2.3	±	1.3

Note: Values are mean ± SD; Y-PHV, year from peak height velocity; MVPA, days of moderate-to-vigorous intensity physical activity over 60 min/day.

**Table 2:** Changes in physical fitness after training intervention.

	Control Group			HIIT Group			ANOVA *p*		
	Pre	Post	Δ (95% CI)	Pre	Post	Δ (95% CI)	Time	Group	Group × Time
20mSR	55.66 ± 19.61	54.45 ± 19.03	−1.21 [−4.29 to 1.88]	42.33 ± 17.01	45.48 ± 20.36 *	3.15 [0.16 to 6.13]	0.359	0.030	0.043
SU	18.41 ± 4.95	18.97 ± 5.20	0.55 [−0.16 to 1.26]	17.22 ± 6.15	19.19 ± 6.25 *	1.96 [0.83 to 3.10]	<0.001	0.743	0.032
SLJ	157.0 ± 22.7	155.7 ± 21.1	−1.29 [−4.29 to 1.71]	145.4 ± 19.5	148.2 ± 19.8	2.73 [-0.30 to 5.76]	0.491	0.089	0.058

Note: Values are mean ± SD; 95% CI, 95% confidence interval; 20mSR, 20 m shuttle run; SU, sit-ups; SLJ, standing long jump; Bold texts indicate significance (*p* < 0.05); * *p* < 0.05, significant difference within group.

**Table 3:** Changes in the digit span test after training intervention.

	Control Group			HIIT Group			ANOVA *p*		
	Pre	Post	Δ (95% CI)	Pre	Post	Δ (95% CI)	Time	Group	Group × Time
DFS									
Score	12.41 ± 2.40	12.97 ± 2.73	0.55 [0.08 to 1.02]	11.93 ± 2.38	13.19 ± 3.00	1.26 [0.52 to 2.00]	<0.001	0.843	0.099
MS	5.31 ± 1.04	5.38 ± 0.94	0.07 [−0.16 to 0.29]	5.19 ± 0.92	5.52 ± 1.19	0.33 [-0.03 to 0.70]	0.055	0.905	0.204
DBS									
Score	8.79 ± 2.47	8.86 ± 2.61	0.07 [−0.59 to 0.73]	7.85 ± 2.51	8.96 ± 2.72 *	1.11 [0.31 to 1.91]	0.023	0.516	0.043
MS	4.14 ± 1.03	4.14 ± 0.95	0.00 [−0.37 to 0.37]	3.59 ± 1.01	4.07 ± 0.96 *	0.48 [0.13 to 0.83]	0.058	0.196	0.058

Note: Values are mean ± SD; 95% CI, 95% confidence interval; DFS, digit forward span; DBS, digit backward test; MS, maximum span; Bold texts indicate significance (*p* < 0.05); * *p* < 0.05, significant difference within group.

**Table 4:** Changes in the Tower of Hanoi test after training intervention.

	Control Group			HIIT Group			ANOVA *p*		
	Pre	Post	Δ (95% CI)	Pre	Post	Δ (95% CI)	Time	Group	Group × Time
Three-disk									
Time	74.9 ± 68.2	27.7 ± 13.5	−45.5 [−72.1 to −19.0]	98.4 ± 102.5	21.1 ± 8.7	−77.2 [−117.6 to −36.9]	<0.001	0.488	0.204
Number	13.2 ± 9.2	10.4 ± 3.0	−2.7 [−6.5 to 1.1]	14.1 ± 6.3	9.7 ± 2.7	−4.4 [−6.9 to −2.0]	0.003	0.918	0.478
Four-disk									
Time	206.7 ± 155.4	69.9 ± 48.3	−132.1 [−189.0 to −75.1]	136.7 ± 117.4	60.6 ± 24.7	−76.1 [−121.7 to −30.4]	<0.001	0.063	0.102
Number	35.6 ± 15.2	25.1 ± 12.3	−10.1 [−17.0 to -3.3]	29.1 ± 13.6	26.0 ± 8.8	−3.1 [−9.6 to 3.4]	0.006	0.287	0.120
Five-disk									
Time	151.5 ± 21.0	159.9 ± 145.3	8.3 [−56.9 to 73.4]	157.1 ± 125.3	98.2 ± 57.6	−58.9 [−114.5 to −3.2]	0.248	0.204	0.126
Number	39.5 ± 39.5	49.4 ± 33.2	9.8 [−5.6 to 25.3]	49.8 ± 38.2	41.3 ± 19.6	−8.5 [−25.7 to 8.7]	0.905	0.851	0.115

Note: Values are mean ± SD; 95% CI, 95% confidence interval; Bold texts indicate significance (*p* < 0.05).
